# The Quality of Red Bell Pepper Subjected to Freeze-Drying Preceded by Traditional and Novel Pretreatment

**DOI:** 10.3390/foods10020226

**Published:** 2021-01-22

**Authors:** Katarzyna Rybak, Artur Wiktor, Dorota Witrowa-Rajchert, Oleksii Parniakov, Małgorzata Nowacka

**Affiliations:** 1 Department of Food Engineering and Process Management, Institute of Food Sciences, Warsaw University of Life Sciences—SGGW, 02-787 Warsaw, Poland; artur_wiktor@sggw.edu.pl (A.W.); dorota_witrowa_rajchert@sggw.edu.pl (D.W.-R.); malgorzata_nowacka@sggw.edu.pl (M.N.); 2 Elea Vertriebs- und Vermarktungsgesellschaft mbH, Prof. von Klitzing Str. 9, 49610 Quakenbrück, Germany; o.parniakov@elea-technology.com

**Keywords:** red bell pepper, blanching, pulsed electric field, ultrasound, hybrid treatment, shock freezing, vacuum freezing, freeze-drying

## Abstract

It has been demonstrated previously in the literature that utilization of PEF or a combination of a pulsed electric field (PEF) and ultrasounds (US) can facilitate dehydration processes and improve the quality of dried products even better than the application of thermal methods such as blanching. The aim of the study was to evaluate the quality of red bell pepper subjected to freeze-drying preceded by blanching or PEF or US treatment applied in a single and combined mode. Furthermore, the freeze-drying was preceded by shock freezing or vacuum freezing performed inside the freeze-dryer as a result of pressure drop during the first stage of freeze-drying. All of the analyzed technological variants enhanced the drying kinetics when compared to the intact material. Freeze-dried bell pepper subjected to non-thermal pretreatment exhibited higher vitamin C, total phenolic and carotenoids content than blanched material despite the fact that blanching reduced drying time the most compared to all other analyzed methods.

## 1. Introduction

Red bell pepper is a very popular vegetable, which can be eaten raw, cooked, as a pickle, paste, or sauce. As a result of its high rate of perishability, it is processed in order to extend its shelf life. In the food industry, red bell pepper is usually added in dried form as a spice or flavor ingredient [1,2]. Furthermore, bell pepper and its extracted compounds can be used as a coloring agent for food and cosmetics [3,4], pharmaceuticals, and nutraceuticals [5]. However, nowadays, dried fruits and vegetables consumed as a snack are becoming highly popular [6,7,8].

Drying is the oldest method of food preservation and one of the most frequently used processing techniques that enable fruits and vegetable surplus to be handled during its season [9,10]. The most often utilized method of drying is convective drying, but the quality of a product usually is not satisfactory due to the loss of thermolabile compounds and structural changes, which occur during an elongated exposure of the plant tissue to high temperatures [7,11,12]. Furthermore, drying is considered to be one of the most energy-consuming processes due to the change in the state of water from liquid to gas phase. It is estimated that drying uses between 12 and 20% of the total energy consumed by the food industry [13]. To maintain the high quality of the dried fruits and vegetables freeze-drying (lyophilization) process can be applied. This process is known as the best method for preserving the nutritional value, color, bioactive compounds as well as structure of raw material due to low temperature, reduced pressure, and lack of air during the process [4,8,14]. However, freeze-drying is a very long and expensive process. Furthermore, freeze-drying requires the product to be frozen in advance, which also increases costs and energy consumption [6]. However, there are solutions such as vacuum freezing [14], which reduce the time and cost of freeze-drying [15]. The preferred type of freezing is quick and shock freezing due to the small and spherical ice crystals formed in this case, which are present both in the intercellular spaces and in the cells of the plant tissue. Due to the fact that the size of the ice crystals is small, they do not disrupt the cell structure, and this results in a better quality of the final product. However, as a result of slow freezing, large hexagonal ice crystals are formed, which has the ability to expand, and by increasing their volume, they destroy cells, causing an unfavorable change in the texture of the product and the loss of valuable compounds [15].

Due to the growing consumer interest in high-quality fruit and vegetables, including taste, color, consistency and nutritional properties, as well as products with a “clean label” claim (without food additives and preservatives) [7], the rapid development of non-thermal techniques in food processing is observed. The techniques such as pulsed electric fields (PEF) and ultrasounds (US) led to a higher or similar quality in comparison to traditional production, but with lower costs. In the last few years, these non-thermal techniques have been carefully studied by several researchers [16,17,18,19], and their results show there have been promising treatment methods to reduce the time of drying processes, which influence cost and energy saving. The non-thermal treatments are considered sustainable and environmentally friendly unit operations [17,19,20,21,22].

Both technologies, PEF and US, are used to modify the internal structure of the material, which resulted in a greater intensity of the water evaporation during the drying process from plant material tissue [17,23]. However, the mechanism of these technologies is different. PEF affects the cell membrane permeability due to electroporation phenomena caused by the application of very short electrical pulses with high voltage. The effect of PEF application is the rupture of cellular structure and loss of cell membrane continuity, which can intensify the mass and/or heat transfer-based unit operations [24,25]. For US treatment, the high power ultrasound is used, where two basic phenomena such as the “sponge effect” and cavitation effect can be distinguished [21,22]. The “sponge effect” depends on squeezing and releasing the material repetitively due to the acoustic wave which goes through it. The cavitation results in creating, increasing and then collapsing of gas bubbles, which is related to rapid local growth of temperature and pressure in the material, and as a consequence, it may influence the formation of microscopic channels [26,27]. These phenomena and processes of sonication led to the modification in the structure of a plant tissue [28] and similarly to PEF, which caused intensifying processes based on mass or heat exchange [17]. For example, sonication of red bell pepper strips in brine solution results in an increase in total solids and water loss, as well as the softening of tissue [29]. Furthermore, as described by Fauster et al. [16], PEF treatment used prior to freeze-drying reduces the shrinkage around 30% in comparison to intact red bell pepper.

However, not only the physical changes occur during non-thermal processing. Chemical and biological changes in the plant tissue take place as well, which influence modification of the final quality product. The improvement of product quality is associated with the proper selection of processing conditions [30,31], which frequently requires conducting much research to optimize the process. Nevertheless, in recent times, hybrid methods are often applied for drying to obtain a better quality of the material. The hybrid method very often combines several pretreatments in specific sequences [12,32] with one or more subsequent drying processes [33]. As a result, it is possible to create the chemical and physical properties of the dried material and eliminate the disadvantages of some drying process techniques, thereby obtaining high-quality products. For example, Nowacka et al. [12] and Wiktor et al. [34] obtained better quality of dried cranberries, and they noticed the increase of effective water diffusivity during convective drying [34] or microwave-vacuum drying [12], which was treated before with hybrid methods of blanching, ultrasound, pulsed electric field, and osmotic dehydration.

Therefore, the aim of the study was to evaluate the quality of red bell pepper subjected to freeze-drying preceded by traditional (blanching in water and steam) and novel pretreatment in a single and hybrid configuration with the use of ultrasound and pulsed electric field. Furthermore, the freeze-drying was preceded by shock freezing or vacuum freezing performed inside the freeze-dryer.

## 2. Materials and Methods

### 2.1. Material

Red bell pepper (*Capsicum annuum* L.) was bought from a local market at Bronisze (Poland). Before the experiment, the vegetables were stored under refrigerated conditions (temperature 5 °C, relative humidity 95%) in darkness. Before processing, the material was washed with cold distilled water and dried with filter paper. Then the seed socket which formed the red bell pepper was removed, and the material was cut into 2 × 4 cm pieces.

### 2.2. Experimental Design

The samples of the red bell pepper underwent traditional treatment such as blanching in water (BL_W) and steam (BL_S), novel single treatment using 30 min of sonication (US) or pulsed electric field treatment with an energy input of 1 (PEF_1) and 3 kJ/kg (PEF_3) or novel hybrid treatment where the combination of single methods was applied (PEF+US, US+PEF) using the same parameters of treatment similar to the novel single treatment. Each pretreatment was conducted at least three times. After pretreatment, samples were subjected to shock freezing (L) and/or vacuum freezing (L_VF). Then the samples were dried using the lyophilization method (2 repetitions). The parameters of the sample treatment are shown in Table 1. More details in the pretreatment, freezing and drying procedures were described in the following chapters.

The changes in the porosity (2 repetitions), color as saturation and total color difference (6 repetitions), sugars content (3 repetitions), and bioactive compounds as total phenolic content, total carotenoids content, vitamin C content, and antioxidant activity (3 repetitions) of the freeze-dried red bell pepper, which previously undergone the shock freezing (L) or vacuum freezing (L_VF) and earlier described treatment, were analyzed.

### 2.3. Traditional and Novel Treatment

#### 2.3.1. Traditional Treatment

Blanching was performed using either hot water or steam. In both cases, a container with tap water at 98 °C was used. The material was placed in a container or on a sieve above the container, where the ratio of water to material was 2:1. The process lasted 3 min, and the system was manually mixed every 15 s. After the process, the material was cooled for 15 s with cold potable water. The paper towel was used to remove excess water from the red bell pepper. Blanching was performed in triplicate.

#### 2.3.2. Novel Treatment

Sonication, pulsed electric field and hybrid methods of treatment, which were a combination of both, were used as novel treatments.

##### Sonication Treatment (US)

The sonication process (US) was carried out with the immersion method. The cut material was placed directly in a 25 × 15 × 10 cm ultrasonic bath model MKD-3 (MKD Ultrasonics, Warsaw, Poland), which made use of 300 W of an ultrasonic power generator and at a frequency of 21 kHz. The ultrasonic bath was filled with tap water (temperature of 20.7 ± 1.2 °C) while maintaining the mass ratio of liquid to material 4:1. After 30 min, the material was thrown onto a sieve to separate the liquid and dried with filter paper. Sonication was performed in triplicate.

##### Pulsed Electric Field Treatment (PEF)

The pulse electric field (PEF) treatment was conducted using a pilot-scale PEF system (Elea Vertriebs- und Vermarktungsgesellschaft mbHVer, Quakenbrück, Germany). The maximum voltage possible to be generated by the device was equal to 30 kV. The system delivered the exponential decay pulses at a frequency of 2 Hz with a monopolar signal and a width of 40 ms. Peppers weighing approximately 200 ± 5 g were placed in the treatment chamber with electrodes made of stainless steel, which was poured over with tap water (conductivity σ = 220 μS/cm and temperature T = 21 ± 1 °C), maintaining the material to water ratio of 1:24. The gap between electrodes was equal to 28 cm. The specific energy consumption (kJ/kg) was in the range of 1–3 kJ/kg, and it was modified by adjusting the number of delivered pulses. All experiments were done using the same electric field strength of 1.07 kV/cm. After the treatment, the material was blotted using filter paper. The technological experiments were done in triplicate.

##### Hybrid Treatment (US)

The hybrid method depended on subsequent utilization of PEF or US in different sequences—PEF followed by US or US followed by PEF—as described in detail in Table 1. The experiments were repeated three times.

### 2.4. Freeze-Drying (Lyophilization)

For shock freezing, the fresh and pretreated material was placed on metal trays and placed in a blast freezer (Shock Freezer HCM 51.20, Irinox, Treviso, Italy) at −40 °C for 4 h. After freezing, the material was transferred directly to a freeze dryer, where the water removal process was carried out. Some of the samples subjected to PEF and hybrid methods were placed directly in the freeze dryer chamber without pre-shock freezing. Lyophilization was performed in a laboratory sublimation dryer (Gamma 1-16 LSC, Martin Christ Gefriertrocknungsanlagen GmbH, Osterode am Harz, Germany). The shelf temperature was set at 40 °C, and the pressure was 0.630 mbar. The condenser temperature was kept constant during the whole process at −55 °C. The shelf load was 2.86 ± 0.09 kg/m^2^. Drying was carried out to achieve a constant mass by the material (the mass of the samples was measured automatically every 15 min). In turn, drying time was determined as the time required for the sample to obtain a moisture ratio of 0.1 (Table 2) as calculated by the equation (Equation (1)):

(1) $$MR=u_{\tau }/u_{0},$$

where u_0_ is the initial moisture content (kg H_2_O/kg dry matter (dm)), and u_τ_ is the moisture content at τ moment of the drying (kg H_2_O/kg dm). Freeze-dried material without any pretreatment (UNTR) was used as a control sample. It was not possible to performed vacuum freezing for untreated and US-treated samples (samples did not freeze due to pressure drop).

The material was taken out of the freeze-dryer chamber after the process has finished, packed into PET12/Al8/PE100 foils refer to the 3 laminated layers that is combined together in the outer-to-inner order of polyethylene (thickness: 12 microns), aluminum (thickness: 8 microns) and polyamide (thickness: 100 microns) with a high barrier against water vapor and gases completely impervious to light, pouches, sealed and stored at room temperature until needed for analysis. The process was conducted at least 2 times.

### 2.5. Quality of Freeze-Dried Red Bell Pepper

#### 2.5.1. Porosity

The porosity of freeze-dried bell pepper was calculated according to the method described by Ciurzyńska and Lenart [35]. The apparent geometric density (ρ_s_) of the material was determined using cleaned sea sand (Chemsolve, Witko, Łódź, Poland) with a grain size of 0.1–0.3 mm. The weighed sample was placed in a glass cylinder and covered with sand to a volume of 25 mL. The method was used to calculate the volume of the sample, including pores. Apparent geometric density was calculated from the ratio of weight to total sample volume.

The apparent helium density (ρ_d_) of the material was determined using a helium gas pycnometer stereopycnometer (Quantachrome, Anton Paar Ltd., St. Albans, UK). The weighed material was placed in a measuring cell with a volume of 25 cm^3^. The chamber in the device was filled with helium, which penetrated the free spaces in the sample, displacing air from it. The analysis was performed in triplicate.

The total porosity, including open and closed pores, was calculated from the formula:

(2) $$P\left[\%\right]=\left(1-\varrho_{d}/\varrho_{s}\right)100\%,$$

#### 2.5.2. Color of Freeze-Dried Red Bell Pepper

For the color measurement, the dried products were crushed using a laboratory mill (IKA A11 basic, IKA Werke GmbH and Co. KG, Staufen, Germany). The powder was placed in a transparent dish. The measurement was performed using a colorimeter (Chroma Meter Konica-Minolta CR-5, Osaka, Japan) in the CIE L* a* b* system where the main parameters are defined as L*—brightness, with the values 0–100% (black-white), a*—greenness-redness, b*—blueness-yellowness. The light source was a standard illuminant D65, measurement geometry de:8° and 2° Standard Observer. White and black tiles were used to calibrate the device. The measurement was performed in 6 replications. Based on the values of the L*, a* and b* parameters, saturation (C*) and the total color difference (ΔE), which indicate the magnitude of the color difference between a raw red bell pepper and dried materials, were calculated with the following equations [27]:

(3) $$C^{*}=\sqrt{{\left(a^{*}\right)}^{2}+{\left(b^{*}\right)}^{2}},$$

(4) $$\Delta E=\sqrt{{\left(\Delta L^{*}\right)}^{2}+{\left(\Delta a^{*}\right)}^{2}+{\left(\Delta b^{*}\right)}^{2}},$$

where ΔL*, Δa*, Δb* are the change of L*, a* and b* parameter between freeze-dried red bell pepper preceded by different pretreatment and shock freezing (L) or vacuum freezing (L_VF) and fresh red bell pepper.

#### 2.5.3. Photographs of the Freeze-Dried Material

The images of the surface of dried peppers were taken using a computer vision system (CVS). The CVS was equipped with a light source, color digital camera (CDC) and image processing software. The dried material was put in a dark box (without external light). The photos were taken with a Nikon D7000 digital camera (Nikon, Tokyo, Japan) with a 105 mm lens positioned vertically above on a sample at a distance of 100 cm; the light source consisted of four fluorescent lamps reduced at an angle of 45°, emitting daylight at a temperature of 6500 K. Photos were saved in JPG format.

#### 2.5.4. Total Sugars Content (TSC)

The TSC was measured using a high-performance liquid chromatograph (HPLC) with a refractive index detector [12,36]. The system consisted of a quaternary pump (Waters 515, Milford, MA, USA), an autosampler (Waters 717, Milford, MA, USA), Waters Sugar Pak I column 300 × 6.5 mm with Sugar-Pak, precolumn and detector (Waters 2414, Milford, MA, USA). The material was grounded in an analytical mill, 0.2 g of the powder was weighed into centrifuge tubes and poured up to 25 mL with 80 °C Milli-Q redistilled water. Extraction was performed on a mechanical stirrer (Shaker Multi Reax; Heidolph Instruments, Schwabach, Germany) for 4 h at room temperature. The solution was centrifuged, the supernatant was passed through a 0.22 µm PTFE (polytetrafluoroethylene) syringe filter and placed in a cooled autosampler. The Milli-Q redistilled water constituted the mobile phase, the flow rate of which was 0.6 mL/min. 10 µl of the solution was dispensed onto the column. The thermostatic temperature of the column and the detector was 90 °C and 50 °C, respectively. The TSC was calculated on the basis of calibration curves for the standards of the individual sugars: glucose, fructose, sucrose (Sigma-Aldrich, Steinheim, Germany). The result is given as the sum of the listed substances. The measurements were conducted three times.

#### 2.5.5. Total Phenolic Content (TPC)

The Folin–Ciocâlteu (F–C) method was utilized to determine the total polyphenols content [12]. The dried material was grounded in an analytical mill (IKA A11 basic, IKA Werke GmbH & Co. KG, Staufen, Germany), and 0.3 g of the powder was weighed into glass beakers, adding 20 mL of 80% ethanol solution (Avantor Performance Materials, Gliwice, Poland) [23]. The solution was slightly heated for 3 min, not allowing it to boil, filtered and made up to 50 mL. 0.18 mL of extract was placed in glass tubes and diluted with 4.92 mL of distilled water; afterward, 0.3 mL of F–C reagent (Merck, Darmstadt, Germany) was added and mixed. After 3 min, the pH of the solution was changed by adding and mixing 0.6 mL of supersaturated sodium carbonate solution (Avantor Performance Materials, Gliwice, Poland). The incubation was carried out in the darkness at 25 °C for an hour. The absorbance of the solutions was measured using a spectrophotometer (Spectronic 200; Thermo Fisher Scientific Inc., Waltham, MA, USA) at a wavelength of 750 nm against a blank sample. The content of polyphenols was calculated as gallic acid equivalent (mg GAE/100 g dm) with the calibration curve in the range of 1–5 mg/mL. The analysis was conducted three times.

#### 2.5.6. Total Carotenoids Content (TCC)

The modified Polish Standard PN-EN 12136:2000 [37] was used to evaluate the TCC of red bell pepper samples. This method is based on spectrophotometric measurement. 0.2 g of the ground sample was weighed into a centrifuge tube with the addition of distilled water (20 mL) and Carrez I and II solutions (each of 1 mL) (VWR Chemicals BDH Prolabo, Leuven, Belgium). After mixing on vortex, the mixture was centrifuged (5 min at 2000× g). The colorless solution was removed, and the residue was extracted with 3 portions of 20 mL of acetone. The solvent was collected into a lab funnel and shaken with 40 mL of petroleum ether. For faster phase separation, 10 mL of distilled water was added. The acetone–water phase was discarded, while the remaining ether solution was poured into a centrifuge tube containing 1.5 g of anhydrous sodium sulfate and centrifuged. The centrifugation solution was poured into a flask and made up to 100 mL with a solvent. The absorbance of the colored solutions was measured at 450 nm (Spectronic 200; Thermo Fisher Scientific Inc., Waltham, MA, USA). The TCC was determined on the basis of the following equation:

(5) $$TCC\left(mg\beta -\mathrm{carotene}/100g\;dm\right)=A_{450}\times 10^{5}\times m_{2}/A_{1\mathrm{cm}}^{1\%}\times m_{1}\times dm,$$

where A_450_-Absorbance; m_2_ is total extract weight; A^1%^_1 cm_ is β-carotene extinction coefficient in petroleum ether (2592); m_1_ is sample weight; dm is dry matter.

The analysis was conducted in triplicate.

#### 2.5.7. Vitamin C Content

The detection of L-ascorbic acid was carried out using an ultra-performance liquid chromatograph UPLC (WATERS Acquity H-Class, Milford, MA, USA) with a photodiode detector [38]. The dry material was crushed using an analytical mill (IKA-Labortechnik, Staufen, Germany). 0.1 g of the sample was weighed into a centrifuge tube, adding 50 mL of extraction reagent (3% metaphosphoric acid, 8% acetic acid, 1 mM ethylenediaminetetraacetic acid (EDTA)—VWR Chemicals BDH Prolabo, Leuven, Belgium) at around 4 °C. The mixture was extracted with the mechanical stirrer (Shaker Multi Reax; Heidolph Instruments, Schwabach, Germany) for 10 min without light and centrifuged (5 min, 6000 rpm with cooling). The solution, filtered through a syringe filter (0.22 µm, PTFE), was diluted with eluent (1:1) and placed in a chilled autosampler (+4 °C). Then 5 µL of the solution was dosed onto the column. Analyses were conducted using a WATERS Acquity UPLC HSS T3 chromatography column (2.1 × 100 mm, 1.8 µm; Waters, Ireland) with an Acquity UPLC HSS T3-precolumn (2.1 mm ID × 5 mm, 1.8 µm). The column was thermostated at 25 °C. The isocratic mobile phase (redistilled water (Milli-Q) with formic acid of 0.1% (*v/v*)) flow rate was 0.25 mL/min. The spectrum was collected at a wavelength of 245 nm. For quantitative analysis, the L-ascorbic acid analytical standard (VWR Chemicals BDH Prolabo, Leuven, Belgium) was utilized to determine a calibration curve (in the range of 0.005–0.100 mg/mL). The analyses were performed three times.

#### 2.5.8. Antioxidant Activity (DPPH and ABTS Assay)

The ability to inactivate free radicals was determined reacting to the analyzed sample with 1,1-diphenyl-2-picrylhydrazyl radical (DPPH^•^) and 2,2-azinobis (3-ethylbenzothiazoline-6-sulfonate) radical cation (ABTS^•+^) according to Lammerskitten et al. [14]. Ethanolic extracts prepared for the TPC analysis were used to determine antioxidant activity [23]. The measurement was made in triplicate. The results were expressed as the effective concentration of the sample, which can decrease the concentration of DPPH^•^ or ABTS^•+^ by 50%.

##### DPPH Assay

The free radical solution of DPPH^•^ (Sigma-Aldrich, Steinheim, Germany) was formed by dissolving DPPH^•^ in methanol solution and stored for 16 h at 4 °C without light. The solution for measurement was made directly before the assay by diluting with 80% (*v*/*v*) aqueous ethanol solution to obtain a solution with absorbance at 515 nm (control solution: 2 mL DPPH + 2 mL solvent) in the range of 0.680–0.720. Different volumes of sample extracts and 80% ethanol were placed in four glass tubes, obtaining the final extract concentration in a range of 0.03–0.45 mg dm/mL. 2 mL of DPPH^•^ solution was added to each tube, mixed and incubated in darkness for 30 min. Based on the absorbance results of both the control solution and the extract solutions, the percentage of scavenging of free radicals was measured for each solution, which allowed us to determine the extract concentration necessary to neutralize 50% of free radicals.

##### ABTS Assay

The free radical solution was prepared by dissolving in distilled water potassium persulfate (Sigma-Aldrich, Steinheim, Germany) and ABTS (Sigma-Aldrich, Steinheim, Germany) and left for 16 h to obtain 7 mmol/L solutions. As a result of diluting 1 mL of stock solution in 100 mL of 80% (*v/v*) aqueous ethanol solution, the working solution with absorbance in the range of 0.680–0.720 at 734 nm was obtained. Variable volumes of extracts were transferred into four glass test tubes, and 3 mL of free radical solution was added and mixed. The final concentrations of the extracts in the solutions ranged from 0.05 to 0.18 mg/mL. After 6 min of incubation in the absence of light, the absorbance of the control and the extracts solutions were measured. The percent of scavenging of free radicals was calculated for each solution, and the sample concentration resulting in a 50% decrease in the ABTS^•+^ was determined.

### 2.6. Statistical Analysis

The samples obtained in the replicates of the same technological treatment were gathered together, and the sampling for analytical procedures was done by random selection. The evaluation of the results was conducted using TIBCO company software (STATISTICA program, version 13, Palo Alto, CA, USA). For results analysis, the ANOVA procedure was used, and the Tukey’s test was applied. The significance of the variance was set for α = 0.05.

## 3. Results and Discussion

### 3.1. Characterization of Drying Process

The drying time understood as the time needed to reach moisture ratio MR = 0.1, was equal to 665 min for untreated red bell pepper (Table 2). The presence of undamaged structures caused difficulties in the mass exchange process, and the cell membrane resisted the evaporation of water from the cells during the drying process. The treatment used before freeze-drying reduced the drying time in the range of 185 to 515 min. The blanching in water reduced the drying time the most (72%). However, this process negatively influenced the product quality. In addition, Krzykowski et al. [5] noted that the drying time of bell pepper pulp using the lyophilization technique decreased by approximately 30% when blanching was implemented as a pretreatment. The reduction of drying time was probably associated with the great damage of the cellular structure caused by the blanching process [39]. The steam blanching also reduced drying time, but only by 23% in comparison to untreated pepper. Novel treatment—performed using only one method or hybrid mode reduced drying time by 29 to 70%. When samples were subjected to US treatment, the drying lasted 440 min, whereas PEF reduced it ranging from 215 to 470 min. In addition, the hybrid methods of treatment reduced drying time in the range of 200 to 380 min. It is worth emphasizing that drying time of the samples frozen using vacuum method was longer in comparison to variants which were subjected to shock-freezing before water removal. This is most probably associated with the swelling, the eruption of constituent liquids, and formation of the foam, which resists and prevents the diffusion of water vapor. As earlier stated in literature, for the tissue with high sugar content this phenomenon is limiting subsequent processes [15].

### 3.2. Visual Assessment of Freeze-Dried Red Bell Pepper, its Porosity, Saturation (C*) and Total Color Difference (ΔE)

**Figure 1:**
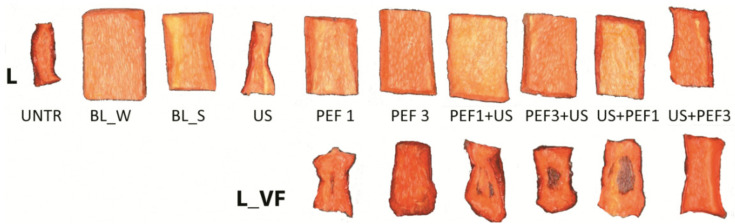
Photo of lyophilized red bell peppers treaded with traditional treatment (BL_W—blanching in water, BL_S—steam blanching), novel single treatment (US—sonication for 30 min, PEF_1—pulsed electric field with energy input 1 kJ/kg, PEF_3—pulsed electric field with energy input 3 kJ/kg) or novel hybrid treatment (PEF+US, US+PEF), preceded by shock freezing (L) and vacuum freezing (L_VF).

Figure 1 shows the freeze-dried red bell peppers obtained as a result of traditional treatment such as blanching in water or in steam, and novel treatment used as a single treatment with US or PEF with different pulsed electric field energy input applied (1 or 3 kJ/kg) and hybrid treatment (PEF+US or US+PEF). The lyophilization process was preceded by shock freezing (L) or vacuum freezing (L_VF). The control sample (UNTR), which was not subjected to any treatment, was achieved using a freeze-drying process with shock freezing. The untreated sample was characterized by high shrinkage and low porosity equal to 27.4 ± 1.7% (Table 3). Similar changes to the untreated one occurred in the sample subjected to sonication, which obtained only 48.8% of porosity. The biggest porosity was found for water (87.9%) and steam (73%) blanched samples, which were associated with the thermal lysis of the internal microstructure.

Blanching is a very popular process used before vegetable processing, which resulted in the reduction of microbial infections and inactivation of the enzymes. During blanching, thermal disruption occurs, and oxygen is removed from intercellular spaces of the tissue [40,41]. The consequence of these processes is high porosity, which was highest for blanching in water (87.9%) from all analyzed samples. In turn, smaller porosity was observed for red bell pepper blanched in steam (73%).

Structure deformations were observed for the freeze-dried samples and preceded with vacuum freezing at the beginning of the lyophilization process, and it was subjected to the novel treatment (Figure 1). The vacuum freezing technique is often destructive to product appearance and structure [15]. These changes are linked to the collapse of macropores, which were filled with water before drying. The porosity is related to structural changes that occur during the dehydration process [42]. On this basis, we can affirm that the freezing process during the lyophilization is not very effective and may cause structure breakdown, which is also visible in the images of samples (Figure 1). The porosity of the freeze-dried preceded with vacuum-freezing material was in the range from 28.8 to 66.4%, whereas samples subjected to the shock freezing exhibited much higher porosity ranging from 69.7% for PEF1 treatment to 82.3% for US+PEF1. Generally, the porosity of the products depends on the freezing rate and the size of the ice crystals [15]. When PEF was applied as a single treatment, the porosity increased. Also, Fauster et al. [16] noticed that volume reduction of bell pepper was lower when the PEF treatment was used before freeze-drying in comparison to the untreated. Similarly, in apple, PEF applied before freeze-drying inhibited shrinkage of the tissue [14]. Furthermore, when novel hybrid treatment was used, regardless of the freezing method applied for freeze-drying, the combination PEF+US resulted in lower porosity than the samples treated with US+PEF. This means that the PEF treatment caused more pronounced structural damages, which are enlarged by the sonication process lasting 30 min. Similar results were noticed by Rajewska and Mierzwa [43] and Wiktor et al. [44], which revealed that PEF treatment plays a major role in hybrid methods. Additionally, the use of the higher specific energy intake for PEF treatment, regardless of application to single or hybrid novel treatment, did not result in significant (*p* > 0.05) changes of sample porosity in comparison with freeze-dried material treated with lower specific energy intake of PEF.

The stability of the color for freeze-dried red bell pepper is the best in comparison to vacuum, infrared, or hot air drying [45]. The saturation C* of freeze-dried red bell pepper is presented in Table 3. The lowest saturation was observed for the intact samples subjected to freeze-drying. All types of treatment resulted in the growth of bell pepper saturation. The lyophilized pepper subjected prior to a single novel method such as PEF treatment and shock freezing resulted in a slight saturation increase. Traditional methods (both water and steam blanching) and sonication caused more visible changes in color saturation. The values of the C* parameter was equal to 61.2, 60.6 and 60.2, for water, steam blanched and sonicated, respectively. Wang et al. [46] noticed that the red color degradation in red pepper followed the first-order reaction kinetics. They observed a 33–59% reduction in red pigment concentration in blanched and hot air-dried red pepper when different parameters were used. Samples treated with hybrid treatment before freeze-drying, regardless of the method of freezing, were characterized by saturation in the range of 57.0 to 63.4. However, only when treatment US+PEF3 was applied, the saturation was significantly higher than in the other samples. Furthermore, it is worth emphasizing that the use of vacuum freezing (L_VF) with novel treatment resulted in a higher value of saturation.

The ΔE (total color difference) was calculated on the basis of L*, a*, and b* color parameters and shows the difference between the dried samples and fresh tissue (Table 2). The value of total color difference greater than 2 indicates color difference, which is noticeable by a naked eye [47]. The ΔE of all untreated freeze-dried material was equal to 36.4, and it was the lowest value among all analyzed variants. Samples subjected to any of the pretreatment before freeze-drying exhibited ΔE in the range of 41.9–52.4. Such results are associated with the consequences caused by each method of pretreatment, such as thermal degradation of microstructure in the case of blanching, electroporation in the case of PEF and long treatment time, as well as leakage in the case of US application. It is also worth emphasizing that ΔE values of samples processed using vacuum freezing were lower in comparison to other variants.

### 3.3. Total Sugars Content of Freeze-Dried Red Bell Pepper

**Figure 2:**
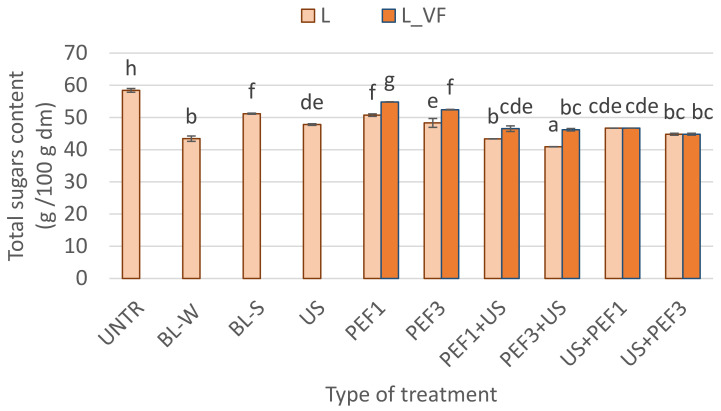
Total sugars content of lyophilized red bell peppers treaded with traditional treatment (BL_W—blanching in water, BL_S—steam blanching), novel single treatment (US—sonication for 30 min, PEF_1—pulsed electric field with energy input 1 kJ/kg, PEF_3—pulsed electric field with energy input 3 kJ/kg) or novel hybrid treatment (PEF+US, US+PEF), preceded by shock freezing (L) and vacuum freezing (L_VF). Data are presented as a mean from 3 repetitions with error bars as a standard deviation (SD), data with different letters differ significantly (Tukey’s HSD, *p* < 0.05).

The total sugars content of lyophilized material exposed to the process of blanching in water and steam, sonication, and PEF, and hybrid treatment, preceded by shock freezing (L) and vacuum freezing (L_VF), is presented in Figure 2. Due to the growing problem of obesity and metabolic syndromes such as diabetes and fast increase in plasma glucose [48], the sugar content is a key parameter in plant-based products, which are considered healthy. The red bell pepper is known for its sweet taste, and it is called the sweet pepper. The major sugars in bell pepper are glucose and fructose, with a higher concentration of fructose than glucose [49]. The untreated freeze-dried material was characterized by 58.4 g/100 g dm, which is in line with reports from the literature. Korkmaz et al. [50] observed that in sun-dried red pepper flakes, the total sugars in amount is 58.7 g/100 g dm. However, the traditional and novel treatment preceded before drying decreased the total sugar content in dried material. Blanching, especially when conducted in water, caused a decrease in the total sugar content by about 12% for steam blanched samples and 26% for water blanching. This effect was connected with leaking the water-soluble solids as sugars into hot water, which are easily dissolved in water. When blanching was conducted in steam, a reduction in sugar depletion was observed. In addition, the reduction of sugar content was observed for samples treated with the novel single and hybrid treatment, which was in the range of 40.9 to 54.8 g/100 g dm. All these processes were conducted in the water at room temperature, and it may have influenced transferring sugars from plant material to the aqueous phase, especially when sonication lasted 30 min. This results in a reduction in the sugar content of the dried product. It should be emphasized that a decrease in sugars may also be linked with cellular rupture, and the cell components are released into the surroundings [51]. The lowest sugar content was noticed for lyophilized red bell pepper obtained when treated by hybrid methods in configuring PEF with the US. The application of PEF treatment may cause structural damages and leakage of intracellular content [43]. This leakage was facilitated by sonication, and sugars were easily transferred to the surrounding water. The sugar content in these samples can be compared to the freeze-dried pepper blanched in water. What is worth emphasizing is that the vacuum freezing was conducted in like manner, or a higher amount of sugars in lyophilized material was observed compared to those subjected to shock freezing before drying. Such results can be associated with the dimensions of ice crystals that are formed during vacuum freezing in comparison to shock freezing. It has been reported that vacuum-induced freezing results in the formation of chimney-like big ice crystals, which can rupture the cellular structure even more and thus improve the extractability of sugars [52,53]. Such explanation sounds reliable when comparing the results obtained for the samples treated using the same pretreatment conditions but different drying methods.

However, the use of an appropriate combination of unconventional PEF+US techniques can reduce the sugar content of dried red pepper even to 30% (L PEF3+US) of the value before drying, similar to a water blanched sample.

### 3.4. Bioactive Compounds of Freeze-Dried Red Bell Pepper

The freeze-drying led to the preservation of valuable compounds in plant material [8]. The lyophilization of the pepper led to obtaining the highest amount of β-carotene, ascorbic acid, and total phenolic content in comparison with other methods of drying such as sun drying, hot drying, and microwave-vacuum drying. However, still, the bioactive compounds are lower in comparison to fresh materials [54]. Thus, the improvements of lyophilization are researched by scientists.

#### 3.4.1. Vitamin C

**Figure 3:**
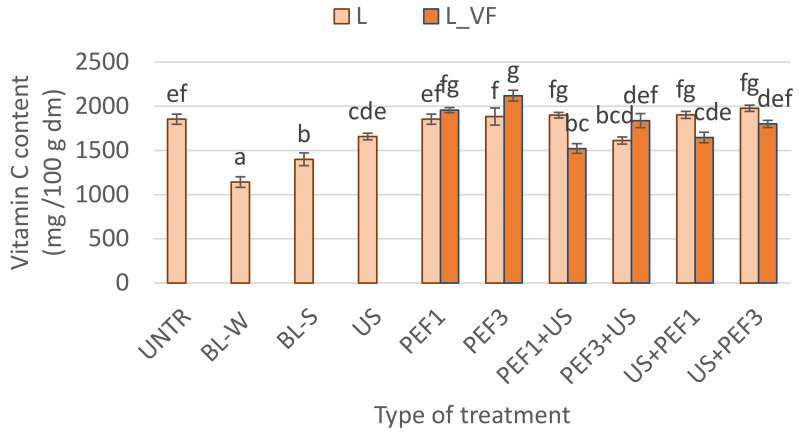
Vitamin C content in lyophilized red bell peppers treaded with traditional treatment (BL_W—blanching in water, BL_S—steam blanching), novel single treatment (US—sonication for 30 min, PEF_1—pulsed electric field with energy input 1 kJ/kg, PEF_3—pulsed electric field with energy input 3 kJ/kg) or novel hybrid treatment (PEF+US, US+PEF), preceded by shock freezing (L) and vacuum freezing (L_VF). Data are presented as a mean from 3 repetitions with error bars as a standard deviation (SD), data with different letters differ significantly (Tukey’s HSD, *p* < 0.05).

The vitamin C content in lyophilized samples is depicted in Figure 3. Vitamin C concentration can be used as the indicator of thermal treatment. If the vitamin C content does not change, it can be stated that there have been no significant changes in other valuable nutrients. Vitamin C is water-soluble, thermolabile and very sensitive to high temperatures [7,55]. Moreover, the presence of light, enzymes, or oxygen also negatively affect vitamin C content [5]. The red bell pepper is known as a good source of vitamin C and contains from 88 to 304 mg/100 g fresh weight (fw) [7,56]. In the present study, it was evident that high temperature resulted in a decrease of vitamin C content by about 38.4 and 24.5% for blanching in water and steam, respectively. Also, a decrease in vitamin C approximately by 21% in red pepper blanched in water followed by freeze-drying was noticed by Krzykowski et al. [5]. Moreover, they found a correlation between redness and vitamin C content. However, greater loss of vitamin C is associated not only with the high temperature but also with flushing out the water-soluble components such as sugars and vitamins from the tissue into the surrounding environment [39,57]. Steam blanching is considered a better method, which preserves higher quality in comparison to water blanching [58]. Furthermore, when single novel treatments were applied before freeze-drying, the alteration of the vitamin C content was observed. For sonication, which was conducted in water for 30 min, the lower vitamin C content by about 11% was noted when compared with the untreated dried sample. It can be stated that sonication also influences the washing out of the vitamin C. Moreover, currently, existing data shows that longer sonication causes the degradation of vitamin C [31], which can be linked with the initiation of numerous reactions by the free radicals created by ultrasound waves [47]. What is interesting, freeze-dried red bell pepper exposed to PEF treatment was similar or even higher by about 2 to 14% content of vitamin C when compared with the untreated dried material. During PEF treatment, the material is also immersed in water, but the treatment was relatively short, i.e., about a few minutes, which resulted in a lower loss of vitamin C. PEF treatment is used as an extraction method, so the permeabilization may influence the better extractability of the bioactive compounds [59,60], in this case, vitamin C. However, also the freezing conducted with a vacuum in the freeze-drying chamber resulted in a higher amount of vitamin C content. For example, Ade-Omowaye et al. [25] noticed that in comparison to the intact sample, vitamin C content decreases from 11 to 24% when high-intensity electric field pulses followed by convective drying were applied. Similarly, when freezing was applied before drying, the 24% degradation of vitamin C occurred.

The use of novel hybrid treatment (PEF+US and US+PEF) remain unchanged or decreased vitamin C as a result of combined treatments. The vitamin C content in these samples was in the range of 1522 to 1977 mg/100 g dm. However, the changes depended on the order of the application of treatment as US and PEF and also by methods of freezing. For freeze-dried pepper subjected to PEF1+US and US+PEF1 and preceded by vacuum freezing, the decrease in vitamin C was observed. In the case of samples marked as PEF3+US, a significantly lower amount of vitamin C content was observed for shock freezing in comparison to freeze-dried pepper without pretreatment. What is worth noting is that all novel treatments retained a higher amount of vitamin C in comparison with traditional treatment methods.

#### 3.4.2. Total Polyphenol Content (TPC)

**Figure 4:**
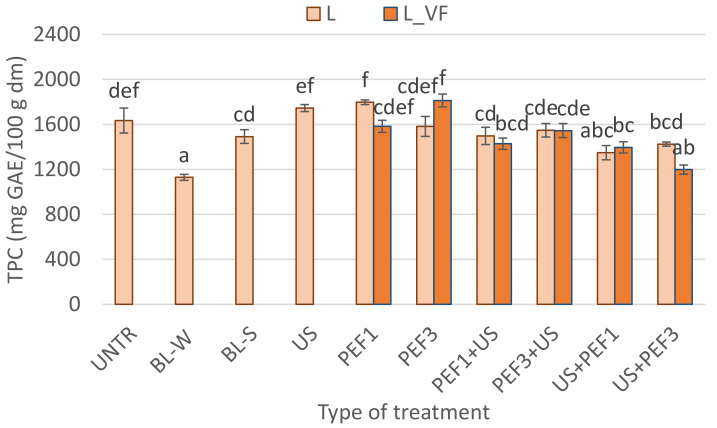
Total phenolic content (TPC) in lyophilized red bell peppers treaded with traditional treatment (BL_W—blanching in water, BL_S—steam blanching), novel single treatment (US—sonication for 30 min, PEF_1—pulsed electric field with energy input 1 kJ/kg, PEF_3—pulsed electric field with energy input 3 kJ/kg) or novel hybrid treatment (PEF+US, US+PEF), preceded by shock freezing (L) and vacuum freezing (L_VF). Data are presented as a mean from 3 repetitions with error bars as a standard deviation (SD), data with different letters differ significantly (Tukey’s HSD, *p* < 0.05).

The total polyphenol content in the lyophilized bell pepper is shown in Figure 4. Untreated freeze-dried red bell pepper contained 1634 ± 111 mg GAE/100 g dm of polyphenols. The temperature had an influence on the polyphenol content in plant material [61]. As expected, the thermal treatment caused degradation of phenols, obtaining lower values by approximately 31% for blanching in water and 9% for steam blanching. For instance, Krzykowski et al. [5] indicated that the water blanching negatively influences the total polyphenols content in freeze-dried pepper pulp. It seems that the dipping plant material in water also has an impact on polyphenols. However, for freeze-dried material earlier subjected to sonication, no significant changes were observed in TPC in comparison with intact dried samples.

The highest amount of polyphenolic compounds was observed for dried samples subjected to PEF treatments. However, these results were not significantly different from those for intact dried material. Moreover, the application of different methods of freezing before drying did not significantly influence the TPC.

A combination of PEF and US used before freeze-drying led to degradation of phenols from 5 to 27% in comparison with untreated freeze-dried material. The lowest value for hybrid treatment was noticed for a sample subjected first to the US and then to PEF with higher specific energy intake. Other samples obtained similar TPC in the range of 1349 to 1547 mg GEA/100 g dm.

#### 3.4.3. Total Carotenoid Content (TCC)

**Figure 5:**
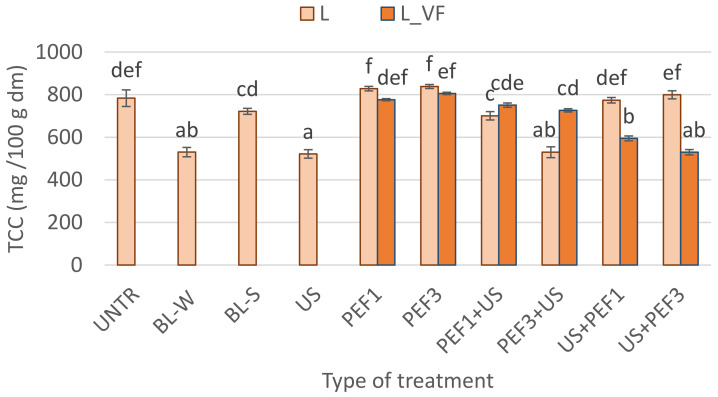
Total carotenoids content (TCC) in lyophilized red bell peppers treaded with traditional treatment (BL_W—blanching in water, BL_S—steam blanching), novel single treatment (US—sonication for 30 min, PEF_1—pulsed electric field with energy input 1 kJ/kg, PEF_3—pulsed electric field with energy input 3 kJ/kg) or novel hybrid treatment (PEF+US, US+PEF), preceded by shock freezing (L) and vacuum freezing (L_VF). Data are presented as a mean from 3 repetitions with error bars as a standard deviation (SD), data with different letters differ significantly (Tukey’s HSD, *p* < 0.05).

Figure 5 presents the total carotenoid content TCC of lyophilized red bell pepper. Carotenoids are sensitive to oxidation [62,63] and elevated temperature [64]. Thus, the application of a thermal treatment, especially blanching in water, reduced the amount of the carotenoids by about 32% (Figure 5). Similarly, Ahmed et al. [64] observed degradation of carotenoids for blanched papaya puree at 70, 80, 90, and 105 °C and stated that a decrease in carotenoids followed first-order reaction kinetics. It is interesting because it is known that generally, carotenoids are hydrophobic antioxidants, which are insoluble in water [65].

The application of US to red bell pepper resulted in a decrease in carotenoids content. As indicated in the literature, sonication causes structural changes, creating microchannels, which speed up the mass transfer, as well as free radicals may be formed by ultrasound waves [47]. In addition, the acoustic waves influence the form of the carotenoids, which aggregate into crystal forms, and resulted in their degradation during drying [63]. It should be emphasized that the degradation of the structure of plant tissue influences carotenoid bioaccessibility [66,67]. However, longer freeze-drying time may also influence the degradation of carotenoids. Similar to freeze-dried paprika pulp, a longer drying time was observed, while the temperature was lower and thus resulted in lighter color and carotenoids decrease [5].

Freeze-dried material preceded by PEF, regardless of the used specific energy intake and methods of freezing, resulted in a carotenoids content similar or higher (by 3–7%) than untreated freeze-dried material. Zhang et al. [66] noted that the hot air drying caused the cell wall disruption, which resulted in a higher release of carotenoid in carrot and yellow bell pepper. PEF treatment can also improve the carotenoid extractability due to the disintegration of the cells in the tissue as a result of the electroporation phenomenon [25,67]. This leads to better extractability of carotenoids, and because of that, higher carotenoids content was observed in the freeze-dried bell pepper. In turn, a hybrid treatment where the US and PEF treatment in different combinations were used, unchanged or caused a decrease of carotenoids content, in comparison with the intact freeze-dried red bell pepper. This means that the PEF1+US, PEF3+US treatment with vacuum freezing, and US+PEF1, US+PEF3 with shock freezing allowed to keep the TCC. It can be stated that the sequence of the hybrid treatment and freezing method is important in the case of bioactive compounds. As previously mentioned, the combination of novel treatment as PEF and US may be associated with different mechanisms connected to the influence of enzymes, structural changes, better extractability, and color changes [53].

#### 3.4.4. Antioxidant Activity

**Figure 6:**
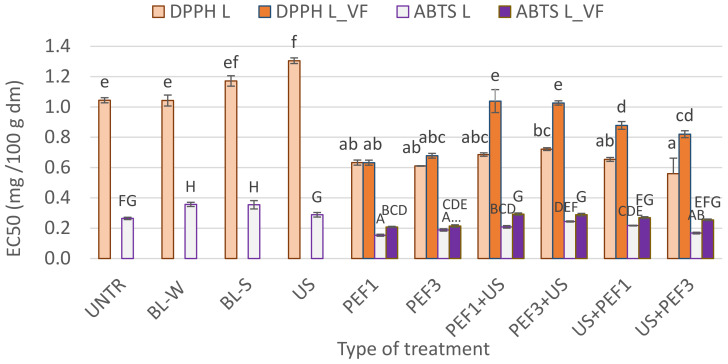
Antioxidant activity expressed as EC50 (the effective concentration of the sample, which can decrease the concentration of 1,1-diphenyl-2-picrylhydrazyl radical—DPPH^•^ or 2,2-azinobis (3-ethylbenzothiazoline-6-sulfonate) radical cation—ABTS^•+^ by 50%) of lyophilized red bell peppers treaded with traditional treatment (BL_W—blanching in water, BL_S—steam blanching), novel single treatment (US—sonication for 30 min, PEF_1—pulsed electric field with energy input 1 kJ/kg, PEF_3—pulsed electric field with energy input 3 kJ/kg) or novel hybrid treatment (PEF+US, US+PEF), preceded by shock freezing (L) and vacuum freezing (L_VF). Data are presented as a mean from 3 repetitions with error bars as a standard deviation (SD), data with different letters lowercase and uppercase differ significantly for L and L_VF, respectively (Tukey’s HSD, *p* < 0.05).

In the present study, antioxidant activity was expressed as an EC50 coefficient, which means that a higher value represents lower antioxidant activity. Thus, EC50 shows the concentration of dry matter, which is capable of scavenging 50% of the DPPH or ABTS radical. For intact freeze-dried samples, DPPH and ABTS EC50 values were equal to 1.04 and 0.26 mg/100 g dm, respectively (Figure 6). The results obtained by DPPH and ABTS assay method allowed us to obtain similar results. It was observed that for both thermal treatment and also for sonication, the antioxidant activity was similar or slightly lower when compared to intact dried material. The use of PEF before lyophilization, especially when the shock freezing was applied, resulting in significantly higher antioxidant activity in comparison with intact freeze-dried bell pepper. The phenomenon of the PEF treatment is associated with the permeabilization process, which leads to the rupture of cell membranes, and this may result in the leakage of intracellular content [14]. In addition, the literature mentions that after PEF treatment, the extractability of bioactive compounds increases.

Antioxidant activity for hybrid treatment used before lyophilization was almost always higher (lower EC50) in comparison to the intact lyophilized sample. However, in this treatment, the freezing process had a significant effect on the EC50. The shock freezing used before lyophilization allowed to keep higher antiradical activity when compared to vacuum freezing in the freeze-drying chamber. It is worth emphasizing that the drying time, which was much longer for samples subjected to vacuum-freezing, may affect the antioxidant activity.

## 4. Conclusions

The use of the traditional and novel pretreatment applied prior to freeze-drying of red bell pepper have an influence on the quality, such as color and bioactive compounds in dried material. In addition, the freezing technic resulted in changes in the time of drying and the quality aspects. The drying time was reduced even by 72% for samples treated with blanching in water. However, in this case, the quality of the lyophilized red bell pepper was the poorest. The better quality and the shorter drying time, in comparison to untreated dried material, had bell pepper subjected to novel single treatment as a pulsed electric field (PEF) and hybrid treatment in configuration PEF+US. The use of PEF with the lower specific energy intake (1 kJ/kg) and/or combined techniques caused a decrease in the freeze-drying time by up to 70%. No synergistic effect of the combination of PEF and US was observed for any of the analyzed variables, except the total sugar content and porosity. Furthermore, the appropriate selection of pretreatment methods led to an increased extractability (and/or retention) of bioactive compounds from the product, which may contribute to their better bioavailability. Moreover, pretreatment with PEF allowed the freeze-drying process to be combined with vacuum freezing without any negative changes in the quality of the product and reduced the processing time in comparison to intact lyophilized material. However, the vacuum freezing resulted in an extension of the drying time when compared to samples subjected to shock freezing.

## Figures and Tables

**Table 1:** Parameters of pretreatment preceded by shock freezing (L) and vacuum freezing (L_VF) of freeze-dried red bell pepper.

Treatment	Description and Treatment Parameters		
Traditional treatment			
UNTR	Untreated sample	-	
BL_W	Blanching in water	98 °C, 3 min	
BL_S	Blanching in steam	98 °C, 3 min	
Novel single treatment			
US	Ultrasound treatment	Sonication time: 30 min, ultrasonic bath with frequency: 21 kHz, ultrasound intensity equal to 3 W/cm^2^	
PEF1	Pulsed electric field treatment	Electric field intensity of 1.07 kV/cm	Specific energy intake: 1.0 kJ/kg
PEF3	Pulsed electric field treatment		Specific energy intake: 3.0 kJ/kg
Novel hybrid treatment (combination of above listed single methods with the same treatment parameters)			
PEF1+US	PEF3+US	US+PEF1	US+PEF3
Freeze-drying (40 °C of shelf temperature, 0.630 mbar of pressure)			
Freezing method		Treated sample	
Shock freezing (L) at −40 °C for 4 h		L (untreated), BL_W, BL_S, US, PEF1, PEF3, PEF1+US, PEF3+US, US+PEF1, US+PEF3	
Vacuum freezing (L_VF) during pressure drop inside the freeze-dryer chamber		PEF1, PEF3, PEF1+US, PEF3+US, US+PEF1, US+PEF3	

**Table 2:** Drying time (to moisture ratio (MR) equal 0.1) of freeze-dried red bell pepper preceded by shock freezing (L) and vacuum freezing (L_VF). Data are presented as a mean from 2 repetitions with a standard deviation (SD).

Treatment	Drying Time (to MR = 0.01) (min)	
Traditional treatment		
	L	
UNTR	665 ± 42 ^g^	
BL_W	185 ± 21 ^a^	
BL_S	515 ±64 ^fg^	
Novel single treatment		
	L	L_VF
US	440 ± 21 ^def^	
PEF1	215 ± 21 ^ab^	470 ± 64 ^ef^
PEF3	275 ± 42 ^abc^	350 ± 42 ^bcde^
Novel hybrid treatment		
	L	L_VF
PEF1+US	200 ± 21 ^ab^	350 ± 21 ^cdef^
PEF3+US	230 ± 42 ^abc^	305 ± 42 ^abcd^
US+PEF1	305 ± 21 ^abcd^	380 ± 21 ^bcde^
US+PEF3	380 ± 21 ^cdef^	290 ± 64 ^abcd^

The same letters indicate the same homogenous groups (α = 0.05, Tukey’s HSD).

**Table 3:** Porosity, saturation (C*) and total color difference (in comparison with fresh material) of lyophilized red bell peppers treaded with traditional treatment (BL_W—blanching in water, BL_—steam blanching), novel single treatment (US—sonication for 30 min, PEF_1—pulsed electric field with energy input 1 kJ/kg, PEF_3—pulsed electric field with energy input 3 kJ/kg) or novel hybrid treatment (PEF+US, US+PEF), preceded by shock freezing (L) and vacuum freezing (L_VF). Data are presented as a mean from 2 (for porosity) and 6 (for color parameters) repetitions with error bars as a standard deviation (SD).

Type of Treatment	Porosity (%)	Saturation (C*) (-)	The Total Color Difference (ΔE) (-)
Traditional treatment			
L			
UNTR	27.4 ± 1.7 ^a^	56.6 ± 1.9 ^a^	36.4 ± 2.1 ^a^
BL_W	87.9 ± 4.0 ^f^	61.2 ± 1.2 ^ef^	50.4 ± 1.6 ^cd^
BL_S	73.0± 2.5 ^ef^	60.6 ± 0.1 ^de^	42.3 ± 0.1 ^b^
Novel single treatment			
L			
US	48.8 ± 2.2 ^bc^	60.2 ± 1.2 ^de^	42.8 ± 1.2 ^b^
PEF1	69.7 ± 3.9 ^ef^	58.8 ± 0.4 ^bcd^	50.1 ± 0.5 ^cd^
PEF3	72.7 ± 3.5 ^ef^	57.8 ± 1.9 ^abc^	43.4 ± 1.7 ^b^
L_VF			
PEF1	51.7 ± 5.0 ^g^	58.8 ± 2.4 ^bcd^	41.9 ± 2.0 ^b^
PEF3	46.0 ± 3.0 ^bc^	62.7 ± 0.8 fg	49.3 ± 0.7 ^cd^
Novel hybrid treatment			
L			
PEF1+US	70.6 ± 3.5 ^ef^	58.7 ± 2.1 ^bcd^	48.8 ± 2.3 ^c^
PEF3+US	73.0 ± 4.8 ^ef^	57.0 ± 0.4 ^ab^	50.7 ± 0.6 ^cd^
US+PEF1	82.3 ± 1.1 ^f^	59.9 ± 1.0 ^cde^	50.4 ± 1.0 ^cd^
US+PEF3	80.5 ± 1.9 ^f^	63.4 ± 1.9 ^g^	52.4 ± 1.4 ^d^
L_VF			
PEF1+US	35.6 ± 0.7 ^ab^	60.3 ± 1.8 ^de^	43.7 ± 2.6 ^b^
PEF3+US	28.8 ± 3.3 ^a^	59.7 ± 1.7 ^cde^	43.4 ± 2.0 ^b^
US+PEF1	66.4 ± 1.8 ^e^	58.4 ± 2.1 ^abcd^	44.9 ± 2.9 ^b^
US+PEF3	62.3 ± 4.1 ^de^	63.2 ± 0.7 ^g^	49.8 ± 0.8 ^cd^

The same letters indicate the same homogenous groups (α = 0.05, Tukey’s HSD).

## Data Availability

The data presented in this study are available on request from the corresponding author.

## References

[1] Arslan D., Özcan M. (2011). Dehydration of red bell-pepper (*Capsicum annuum* L.): Change in drying behavior, colour and antioxidant content. Food Bioprod. Process..

[2] Buendía-Moreno L., Soto-Jover S., Ros-Chumillas M., Antolinos-López V., Navarro-Segura L., Sánchez-Martínez M.J., Martínez-Hernández G.B., López-Gómez A. (2020). An innovative active cardboard box for bulk packaging of fresh bell pepper. Postharvest Biol. Technol..

[3] Vengaiah P., Pandey J. (2007). Dehydration kinetics of sweet pepper (Capsicum annum L.). J. Food Eng..

[4] Różyło R. (2020). Recent trends in methods used to obtain natural food colorants by freeze-drying. Trends Food Sci. Technol..

[5] Krzykowski A., Dziki D., Rudy S., Gawlik-Dziki U., Polak R., Biernacka B. (2018). Effect of pre-treatment conditions and freeze-drying temperature on the process kinetics and physicochemical properties of pepper. LWT.

[6] Morais R.M., De Morais R.M.S.C., Dammak I., Bonilla J., Sobral P.J.A., Laguerre J.-C., Afonso M.J., Ramalhosa E. (2018). Functional Dehydrated Foods for Health Preservation. J. Food Qual..

[7] Tylewicz U., Nowacka M., Rybak K., Drozdzal K., Dalla Rosa M., Mozzon M. (2020). Design of Healthy Snack Based on Kiwifruit. Molecules.

[8] Ozcelik M., Ambros S., Morais S.F., Kulozik U. (2020). Storage stability of dried raspberry foam as a snack product: Effect of foam structure and microwave-assisted freeze drying on the stability of plant bioactives and ascorbic acid. J. Food Eng..

[9] Chua K., Chou S. (2003). Low-cost drying methods for developing countries. Trends Food Sci. Technol..

[10] Rybak K., Samborska K., Jedlinska A., Parniakov O., Nowacka M., Witrowa-Rajchert D., Wiktor A. (2020). The impact of pulsed electric field pretreatment of bell pepper on the selected properties of spray dried juice. Innov. Food Sci. Emerg. Technol..

[11] Vega A., Fito P., Andrés A., Lemus R. (2007). Mathematical modeling of hot-air drying kinetics of red bell pepper (var. Lamuyo). J. Food Eng..

[12] Nowacka M., Wiktor A., Anuszewska A., Dadan M., Rybak K., Witrowa-Rajchert D. (2019). The application of unconventional technologies as pulsed electric field, ultrasound and microwave-vacuum drying in the production of dried cranberry snacks. Ultrason. Sonochemistry.

[13] Stojceska V., Atuonwu J., Tassou S.A. (2019). Ohmic and conventional drying of citrus products: Energy efficiency, greenhouse gas emissions and nutritional properties. Energy Procedia.

[14] Lammerskitten A., Wiktor A., Siemer C., Toepfl S., Mykhailyk V., Gondek E., Rybak K., Witrowa-Rajchert D., Parniakov O. (2019). The effects of pulsed electric fields on the quality parameters of freeze-dried apples. J. Food Eng..

[15] Ghio S., Barresi A.A., Rovero G. (2000). A Comparison of Evaporative and Conventional Freezing Prior to Freeze-Drying of Fruits and Vegetables. Food Bioprod. Process..

[16] Fauster T., Giancaterino M., Pittia P., Jaeger H. (2020). Effect of pulsed electric field pretreatment on shrinkage, rehydration capacity and texture of freeze-dried plant materials. LWT.

[17] Witrowa-Rajchert D., Wiktor A., Sledz M., Nowacka M. (2014). Selected Emerging Technologies to Enhance the Drying Process: A Review. Dry. Technol..

[18] Paniwnyk L. (2017). Applications of ultrasound in processing of liquid foods: A review. Ultrason. Sonochemistry.

[19] Barba F.J., Parniakov O., Pereira S.A., Wiktor A., Grimi N., Boussetta N., Saraiva J.A., Raso J., Martin-Belloso O., Witrowa-Rajchert D. (2015). Current applications and new opportunities for the use of pulsed electric fields in food science and industry. Food Res. Int..

[20] Radhakrishnan M., Tiwari B. (2021). Application of Ultrasound in Fruit and Vegetable Processing.

[21] AshokKumar M. (2015). Applications of ultrasound in food and bioprocessing. Ultrason. Sonochemistry.

[22] Knorr D., Zenker M., Heinz V., Lee D.-U. (2004). Applications and potential of ultrasonics in food processing. Trends Food Sci. Technol..

[23] Sledz M., Wiktor A., Nowacka M., Witrowa-Rajchert D. (2017). Drying Kinetics, Microstructure and Antioxidant Properties of Basil Treated by Ultrasound. J. Food Process. Eng..

[24] Lammerskitten A., Mykhailyk V., Wiktor A., Toepfl S., Nowacka M., Bialik M., Czyżewski J., Witrowa-Rajchert D., Parniakov O. (2019). Impact of pulsed electric fields on physical properties of freeze-dried apple tissue. Innov. Food Sci. Emerg. Technol..

[25] Ade-Omowaye B., Taiwo K., Eshtiaghi N., Angersbach A., Knorr D. (2003). Comparative evaluation of the effects of pulsed electric field and freezing on cell membrane permeabilisation and mass transfer during dehydration of red bell peppers. Innov. Food Sci. Emerg. Technol..

[26] Fernandes F.A., Gallão M.I., Rodrigues S. (2009). Effect of osmosis and ultrasound on pineapple cell tissue structure during dehydration. J. Food Eng..

[27] Fijalkowska A., Nowacka M., Witrowa-Rajchert D. (2017). The physical, optical and reconstitution properties of apples subjected to ultrasound before drying. Ital. J. Food Sci..

[28] Pieczywek P.M., Kozioł A., Konopacka D., Cybulska J., Zdunek A. (2017). Changes in cell wall stiffness and microstructure in ultrasonically treated apple. J. Food Eng..

[29] Gabaldón-Leyva C.A., Quintero-Ramos A., Barnard J., Balandrán-Quintana R.R., Talamás-Abbud R., Jiménez-Castro J. (2007). Effect of ultrasound on the mass transfer and physical changes in brine bell pepper at different temperatures. J. Food Eng..

[30] Wiktor A., Sledz M., Nowacka M., Rybak K., Chudoba T., Lojkowski W., Witrowa-Rajchert D. (2015). The impact of pulsed electric field treatment on selected bioactive compound content and color of plant tissue. Innov. Food Sci. Emerg. Technol..

[31] Nowacka M., Fijalkowska A., Dadan M., Rybak K., Wiktor A., Witrowa-Rajchert D. (2018). Effect of ultrasound treatment during osmotic dehydration on bioactive compounds of cranberries. Ultrason.

[32] Wiktor A., Gondek E., Jakubczyk E., Dadan M., Nowacka M., Rybak K., Witrowa-Rajchert D. (2018). Acoustic and mechanical properties of carrot tissue treated by pulsed electric field, ultrasound and combination of both. J. Food Eng..

[33] Júnior E.V.D.S., De Melo L.L., De Medeiros R.A.B., Barros Z.M.P., Azoubel P.M. (2018). Influence of ultrasound and vacuum assisted drying on papaya quality parameters. LWT.

[34] Wiktor A., Nowacka M., Anuszewska A., Rybak K., Dadan M., Witrowa-Rajchert D. (2019). Drying Kinetics and Quality of Dehydrated Cranberries Pretreated by Traditional and Innovative Techniques. J. Food Sci..

[35] Ciurzyńska A., Lenart A. (2016). Effect of the aerated structure on selected properties of freeze-dried hydrocolloid gels. Int. Agrophysics.

[36] El Kossori R.L., Villaume C., El Boustani E., Sauvaire Y., Méjean L. (1998). Composition of pulp, skin and seeds of prickly pears fruit (Opuntia ficus indica sp.). Plant Foods Hum. Nutr..

[37] Polish Standard (2020). Fruit and Vegetable Juice—Total Carotenoids and Carotenoids Fraction Determination (PN-EN 12136).

[38] Spínola V.A.R., Mendes B.R., Câmara J.D.S., Castilho P. (2012). An improved and fast UHPLC-PDA methodology for determination of L-ascorbic and dehydroascorbic acids in fruits and vegetables. Evaluation of degradation rate during storage. Anal. Bioanal. Chem..

[39] Castro S.M., Saraiva J.A., Domingues F.M., Delgadillo I. (2011). Effect of mild pressure treatments and thermal blanching on yellow bell peppers (*Capsicum annuum* L.). LWT.

[40] Sledz M., Wiktor A., Rybak K., Nowacka M., Witrowa-Rajchert D. (2016). The impact of ultrasound and steam blanching pre-treatments on the drying kinetics, energy consumption and selected properties of parsley leaves. Appl. Acoust..

[41] Di Cesare L.F., Forni E., Viscardi D., Nani R.C. (2003). Changes in the Chemical Composition of Basil Caused by Different Drying Procedures. J. Agric. Food Chem..

[42] Donsì G., Ferrari G., Nigro R., Di Matteo P. (1998). Combination of Mild Dehydration and Freeze-Drying Processes to Obtain High Quality Dried Vegetables and Fruits. Food Bioprod. Process..

[43] Rajewska K., Mierzwa D. (2017). Influence of ultrasound on the microstructure of plant tissue. Innov. Food Sci. Emerg. Technol..

[44] Wiktor A., Gondek E., Jakubczyk E., Nowacka M., Dadan M., Fijalkowska A., Witrowa-Rajchert D. (2016). Acoustic emission as a tool to assess the changes induced by pulsed electric field in apple tissue. Innov. Food Sci. Emerg. Technol..

[45] Park J.H., Kim C.S. (2007). The stability of color and antioxidant compounds in paprika (*Capsicum annuum* L.) powder during the drying and storing process. Food Sci. Biotechnol..

[46] Wang J., Yang X.-H., Mujumdar A.S., Fang X.-M., Zhang Q., Zheng Z.-A., Gao Z.-J., Xiao H.-W. (2018). Effects of high-humidity hot air impingement blanching (HHAIB) pretreatment on the change of antioxidant capacity, the degradation kinetics of red pigment, ascorbic acid in dehydrated red peppers during storage. Food Chem..

[47] Tiwari B., Patras A., Brunton N., Cullen P.J., O’Donnell C.P. (2010). Effect of ultrasound processing on anthocyanins and color of red grape juice. Ultrason. Sonochemistry.

[48] Kowalska K., Olejnik A. (2016). Beneficial effects of cranberry in the prevention of obesity and related complications: Metabolic syndrome and diabetes—A review. J. Funct. Foods.

[49] Serrano M., Zapata P.J., Castillo S., Guillén F., Martínez-Romero D., Valero D. (2010). Antioxidant and nutritive constituents during sweet pepper development and ripening are enhanced by nitrophenolate treatments. Food Chem..

[50] Korkmaz A., Hayaloglu A.A., Hayaloglu A.A. (2020). Changes in volatile compounds, sugars and organic acids of different spices of peppers (*Capsicum annuum* L.) during storage. Food Chem..

[51] Karki B., Lamsal B.P., Jung S., Van Leeuwen J. (2010). (Hans); Pometto, A.; Grewell, D.; Khanal, S.K. Enhancing protein and sugar release from defatted soy flakes using ultrasound technology. J. Food Eng..

[52] Kramer M., Sennhenn B., Lee G. (2002). Freeze-drying using vacuum-induced surface freezing. J. Pharm. Sci..

[53] Rybak K., Wiktor A., Witrowa-Rajchert D., Parniakov O., Nowacka M. (2020). The Effect of Traditional and Non-Thermal Treatments on the Bioactive Compounds and Sugars Content of Red Bell Pepper. Molecules.

[54] Maurya V.K., Gothandam K.M., Ranjan V., Shakya A., Pareek S. (2018). Effect of drying methods (microwave vacuum, freeze, hot air and sun drying) on physical, chemical and nutritional attributes of five pepper (*Capsicum annuum* var. *annuum* ) cultivars. J. Sci. Food Agric..

[55] Orikasa T., Koide S., Okamoto S., Imaizumi T., Muramatsu Y., Takeda J.-I., Shiina T., Tagawa A. (2014). Impacts of hot air and vacuum drying on the quality attributes of kiwifruit slices. J. Food Eng..

[56] Castro S.M., Saraiva J.A., Lopes-Da-Silva J.A., Delgadillo I., Van Loey A., Smout C., Hendrickx M. (2008). Effect of thermal blanching and of high pressure treatments on sweet green and red bell pepper fruits (*Capsicum annuum* L.). Food Chem..

[57] Galoburda R., Kuka M., Cakste I., Klava D. (2015). The effect of blanching temperature on the quality of microwave-vacuum dried mushroom Cantharellus cibarius. Agron. Res..

[58] Dadan M., Rybak K., Wiktor A., Nowacka M., Żubernik J., Witrowa-Rajchert D. (2018). Selected chemical composition changes in microwave-convective dried parsley leaves affected by ultrasound and steaming pre-treatments—An optimization approach. Food Chem..

[59] Nowacka M., Tappi S., Wiktor A., Rybak K., Miszczykowska A., Czyzewski J., Drozdzal K., Witrowa-Rajchert D., Tylewicz U. (2019). The Impact of Pulsed Electric Field on the Extraction of Bioactive Compounds from Beetroot. Foods.

[60] Wiktor A., Śledź M., Nowacka M., Chudoba T., Witrowa-Rajchert D. (2014). Pulsed Electric Field Pretreatment for Osmotic Dehydration of Apple Tissue: Experimental and Mathematical Modeling Studies. Dry. Technol..

[61] Deng J., Yang H., Çapanoğlu E., Cao H., Xiao J. (2018). Technological Aspects and Stability of Polyphenols.

[62] Nowacka M., Wedzik M. (2016). Effect of ultrasound treatment on microstructure, colour and carotenoid content in fresh and dried carrot tissue. Appl. Acoust..

[63] Konopacka D., Cybulska J., Zdunek A., Dyki B., Machlańska A., Celejewska K. (2017). The combined effect of ultrasound and enzymatic treatment on the nanostructure, carotenoid retention and sensory properties of ready-to-eat carrot chips. LWT.

[64] Ahmed J., Shivhare U., Sandhu K.S. (2002). Thermal Degradation Kinetics of Carotenoids and Visual Color of Papaya Puree. J. Food Sci..

[65] Háda M., Nagy V., Deli J., Agócs A. (2012). Hydrophilic Carotenoids: Recent Progress. Molecules.

[66] Zhang Z., Wei Q., Nie M., Jiang N., Liu C., Liu C., Li D., Xu L. (2018). Microstructure and bioaccessibility of different carotenoid species as affected by hot air drying: Study on carrot, sweet potato, yellow bell pepper and broccoli. LWT.

[67] Roohinejad S., Everett D.W., Oey I. (2014). Effect of pulsed electric field processing on carotenoid extractability of carrot purée. Int. J. Food Sci. Technol..

